# The Current and Retrospective Intentional Nature Exposure Scales: Development and Factorial Validity

**DOI:** 10.3390/ijerph16224443

**Published:** 2019-11-12

**Authors:** Carly Wood, David Barron, Nina Smyth

**Affiliations:** 1 School of Life Sciences, University of Westminster, London W1W6UW, UK; 2 Centre for Psychological Medicine, Perdana University, 43400 Serdang, Selangor, Malaysia; david@perdanauniversity.edu.my; 3 School of Social Sciences, University of Westminster, London W1W6UW, UK; n.smyth@westminster.ac.uk

**Keywords:** nature, physical activity, green exercise

## Abstract

Both nature exposure and green exercise (GE) can improve health. However, there are no scales examining frequency of engagement; or that consider interaction with nature. There are also no scales assessing these variables during childhood. The aim of this study was to develop a modified (NES-II) and retrospective (RNES-II) version of the Nature Exposure Scale to incorporate GE and to examine their factor structure and reliability. Exploratory factor analysis (EFA) explored the factor structure of the scales; followed by confirmatory factor analysis to confirm the model fit. Fit indices for the one factor five item NES-II and RNES-II models identified by EFA were poor. Use of modification indices resulted in a good model fit; NES-II: χ(5, n = 385) = 2.638; χ_normed_ = 0.879; CFI= 1.000; RMSEA < 0.001 with 90%CI = 0.000–0.082; SRMR = 0.009; AIC = 36.638. RNES-II: χ(2, n = 385) = 7.149; χ_normed_ = 3.574; CFI = 0.995; RMSEA = 0.082 with 90%CI = 0.023–0.151; SRMR = 0.015; AIC = 43.149. Both models demonstrated very good reliability (α = 0.84; 89 respectively). These findings indicate that the scales can be used to assess current and retrospective nature exposure. However, due to the removal of item one, the authors recommend that the scales be named the ‘intentional nature exposure scale’ and ‘retrospective intentional nature exposure scale’.

## 1. Introduction

Exposure to nature, that is “direct physical and/or sensory contact with the natural environment” (e.g., sitting in a natural environment, gardening) [1], can improve mental wellbeing, attention and mood, reduce stress, lower morbidity and reduce cardiovascular disease risk [2,3,4]. These benefits are also derived from contact with natural spaces containing water [5]. Nature exposure also has a beneficial impact on physical activity (PA), promoting participation in and higher intensities of exercise [6,7]. Furthermore, being physically active whilst exposed to nature (so called “green exercise” (GE)) provides additive benefits for health above either PA or nature exposure alone [8,9]. GE can reduce stress and blood pressure, increase self-esteem, mood, and improve heart rate variability [8,10,11]. 

There is also an emerging body of evidence indicating that connectedness to nature, defined as an individual’s “levels of feeling emotionally connected to the natural world” [12], is important for health and might mediate the health benefits derived from nature exposure [13,14]. Thus, public health policies should not only focus on encouraging nature exposure and participation in PA but also on enabling individuals to develop connections with the natural environment. 

In order to allow researchers to further assess the links between nature exposure and health, questionnaires to assess frequency of exposure are required. These questionnaires should not only assess how often an individual is exposed to nature but should specifically encourage individuals to consider any periods of GE and how much notice they take of their surrounding environments; as all of these factors can have an impact on the derived health outcomes [12,15]. Although GPS tracking systems can be used to determine the time an individual spends in natural environments, analysis of this data can be time consuming and complex. Furthermore, these trackers do not enable researchers to determine habitual levels of exposure during selected time periods, but a snapshot of exposure at a set moment in time [16]. They also fail to capture an individual’s level of interaction with their surrounding environments.

To date, there are no scales that measure nature exposure whilst also considering periods of GE and interaction with nature. Largo-Wight, William, Virginia and Weiler [17] developed the Nature Contact Questionnaire; however, this scale only assesses nature exposure in the workplace, with much of the scale being focused on indoor nature such as plants. The Natural Environment Exposure scale [18] examines frequency of exposure to 13 different natural environments. However, the validity of the scale has not been examined, the environments are specific to Australia and there is no consideration of how much notice the individual takes of the environment or whether they participate in any forms of GE when within them.

The Nature Exposure questionnaire [19] examines exposure to nature during every day and non-everyday environments and therefore obtains a measure of exposure to nature across the different areas of an individual’s life. The scale also considers how much notice an individual takes of the natural environments they are exposed to and may therefore tap in to an individual’s level of connection to the natural world. However, the scale does not encourage individuals to consider any periods of GE and the factor structure of the scale has not been confirmed. 

Within the field of environment and health there is also evidence to suggest that nature exposure during childhood can influence adult health outcomes, largely through the development of lifelong healthy habits [18,20,21]. However, there are currently no known scales to assess adults’ childhood nature exposure. 

The primary aim of this study was therefore to develop a modified version of the nature exposure scale to incorporate assessment of nature exposure during PA and to examine the factor structure and internal consistency of both the original Nature Exposure Scale (NES) [19] and the modified version (entitled the Nature Exposure Scale-II; NES-II). The secondary aim of this study was to develop a retrospective version of the NES-II (RNES-II) and to examine its factor structure and internal consistency. 

## 2. Materials and Methods 

### 2.1. Participants and Procedures

Participants were recruited online and via the university student body. Links to the questionnaire were sent out via student blackboard platforms, social media and advertised via the Prolific Academic website (http://prolific.ac); a crowdsourcing internet marketplace that allows individuals to complete academic surveys for monetary compensation. These resources are increasingly being used in psychological studies and provide demographically-diverse and high-quality data [22]. All participants provided informed consent prior to completing the survey and were directed to the Qualtrics website which hosted the survey. Two filler questions were added (“To confirm you are still paying attention please select strongly disagree”) to identify any participants whom were not reading the questionnaire or accurately answering the questions. All responses were anonymous and ethical approval was granted by the University of Westminster research ethics committee prior to commencing the study.

The final sample consisted of 832 participants: 271 were recruited through social media, 57 through student recruitment and 504 through Prolific. Following data screening, 765 participants’ data remained for analysis; including 342 men and 415 women (seven participants gender was unknown) aged 18–92 years (*M* = 33.05, *SD* = 12.05). Most participants (80.4%) were white, while 10.5% were of Asian Ancestry, 2.1% of African American Ancestry and 3.8% of mixed ancestry. A further 2.6% were of some other ethnic background and 0.7% did not wish to reveal their ethnic background. The majority of participants (66.1%) reported that they were in employment, whilst 11.2% were students, 8.6% unemployed, 1.2% retired, and 12.2% ‘other’ including those who were carers or sick/disabled. An additional 0.7% reported that they did not wish to divulge their occupation. 

All participants provided demographic information and completed the NES, NES-II and RNES-II. A sub-set of participants (n = 310) also completed scales to assess wellbeing; depression, stress and anxiety; connectedness to nature and self-esteem to enable the relationship with health outcomes to be examined 

### 2.2. Measures

#### 2.2.1. Nature Exposure Scale (NES)

The NES [19] (Appendix A) is a four-item scale designed to assess “direct physical and/or sensory contact with the natural environment” [19]. Two items are designed to assess exposure to nature in everyday life, and two items to assess exposure outside of everyday environments. However, this factor structure has not been confirmed. Each question on the scale is scored on a 5-point Likert scale (1 = high/a great deal, 5 = low/not much), with higher scores reflecting greater exposure to nature. The scale has previously been demonstrated to have an acceptable level of reliability (α = 0.73). 

#### 2.2.2. Modified Nature Exposure Scale (NES-II)

The authors of this paper developed a revised version of the NES (see Appendix B) which included two additional questions assessing nature exposure during PA. These questions were developed based on the authors’ expertise in GE and to follow the same format as the questions in the NES. The authors postulated that these two items would further enhance the reliability of the NES. Each question is scored on a 5-point Likert scale (1 = high/a great deal, 5 = low/not much); with higher scores representing greater nature exposure. 

#### 2.2.3. Retrospective Nature Exposure Scale (RNES-II)

The questions on the NES-II were revised to ask participants about their experiences of nature and GE during childhood (see Appendix C). For the purpose of this study the authors defined childhood as between the ages of 5–10 years; in line with attendance at primary school. The authors postulated that the structure of the scale would be identical to the NES-II but would simply reflect overall nature exposure during childhood. As per the NES-II the questions were each scored on a 5-point Likert scale (1 = high/a great deal, 5 = low/not much), with higher scores representing greater nature exposure during childhood.

#### 2.2.4. Short Form Warwick Edinburgh Mental Wellbeing Scale (WEMWBS)

The short form WEMWBS is a 7-item positively worded scale which monitors wellbeing in the general population [23]. The scale has five response categories from one (none of the time) to five (all of the time) which are summed to give a single score between 7–35. A higher score represents a better wellbeing. The scale has previously been demonstrated to have a Cronbach alpha score of 0.89 [24]. In the current sample the alpha was 0.86 indicating very good reliability. 

#### 2.2.5. Depression, Anxiety and Stress Scale-21 (DASS-21)

The Depression, Anxiety and Stress Scale-21 is a 21-item scale that is designed to measure the emotional states of depression, anxiety and stress [25]. Each of these sub-scales contain seven items with responses categories from zero (did not apply to me at all) to three (applied to me very much or most of the time). The overall score for each sub-scale is calculated by summing the items and ranges from 0–21; with a higher score representing greater feelings of depression, anxiety or stress. The sub-scales have previously been demonstrated to have reliabilities of 0.88, 0.82 and 0.90 respectively [25]. In the current sample the subscales had Cronbach alphas of 0.91, 0.81 and 0.86 respectively, indicating very good reliability.

#### 2.2.6. Connectedness to Nature Scale (CNS)

The connectedness to nature scale is a measure of an individuals’ trait levels of feeling emotionally connected to the natural world [12]. The scale consists of 14 items with response categories from one (strongly disagree) to five (strongly agree). Three items are reversed scored, with the overall scale score ranging from 14–70. A higher score represents a greater connection to nature. The scale has previously been demonstrated to have a Cronbach alpha of 0.84 [12]. In the current sample the alpha was 0.86 indicating very good reliability.

#### 2.2.7. Rosenberg’s Self-Esteem Scale

Rosenberg’s Self-esteem scale is the most widely used measure of self-esteem and assesses an individual’s feeling towards themselves [26]. The scale consists of ten items, with response categories ranging from one (strongly disagree) to four (strongly agree). Five items are reverse scored, with the overall score ranging from 10–40; and a higher score representing a better self-esteem. Cronbach alpha scores of 0.77 have been demonstrated for the scale [26]. In the current sample the alpha was 0.90 indicating very good reliability.

### 2.3. Statistical Analysis

#### 2.3.1. Exploratory Factor Analysis

Using age and gender matching techniques one half of the data (n = 381) was randomly selected using SPSS statistical analysis software V23 (SPSS Inc. An IBM Company). This data was submitted to principle axis exploratory factor analysis (EFA) to explore the factor structure of the original NES, NES-II and RNES-II. The sample size provided a participant-to-item ratio of 64:1; in excess of the conservative requirement of a 10:1 ratio needed for EFA [27]. Following standard guidelines [28], items were submitted to EFA if they passed standard criteria for item distribution (kurtosis values < 10), average correlation with the other items (items < 0.40 should be removed), and item–total correlation (items should be dropped that are < 0.30). Internal consistency of the scales was assessed using Cronbach alpha to determine whether reliability would have been improved by the removal of any of the items. A Cronbach alpha coefficient of between 0.65 and 0.70 is minimally acceptable, 0.70 to 0.80 respectable, and > 0.80 very good [29]. Items which did not meet criteria or adversely influenced reliability were removed. 

Bartlett’s test of sphericity [30] and the Kaiser–Meyer–Oblim (KMO) measure of sampling adequacy were used to assess factorability of the data [31]. For the data to be deemed suitable for EFA, Bartlett’s test should be significant whilst the KMO index should be above 0.6. 

The number of factors extracted was determined by eigenvalues above 1.0, examination of the scree plot; and the results of parallel analysis when more than one factor was identified. Direct Oblim rotation was used for all scales to allow inter-correlations between factors. Factor loadings were interpreted using Comrey and Lee’s [32] recommendations (i.e., > 0.71 = excellent, > 0.63 = very good, > 0.55 = good, > 0.45 = fair, and > 0.32 = poor).

#### 2.3.2. Confirmatory Factor Analysis

Data from the second half of the sample (n = 385) was used for confirmatory factor analysis (CFA) using AMOS v23. Modelling of the three scales was based on the results of the earlier EFA, and for the original NES the hypothesised model of Francis [19]. The overall fit of each model was assessed using a range of goodness of fit statistics. The normed chi-square model was examined and represents the ratio of χ^2^ to degrees of freedom (called χ _normed_) [33]. A value of 3.0 or less was used to indicate a good fit [31]. The Comparative Fit Index (CFI) was used to determine how well the model compared to a nested baseline model. A value close to or greater than 0.95 indicates an adequate model fit and was therefore used as the cut off value. The Standardised Root Mean Square Residual (SRMR) assessed the mean absolute correlation residual, with a smaller SRMR indicating a better fit. The cut off of < 0.08 recommended by Hu and Bentler [34] was used for this statistic. The Root Mean Square Error of Approximation (RMSEA) was examined to measure the extent to which the models were supported per degree of freedom. RMSEA values close to 0.06 indicate a good fit, with values ranging to 0.10 representing a mediocre fit [31]. Modification indices (MI) were also consulted to improve the accuracy of each model; with both theoretical and statistical sense used to guide model refinement. For the latter a stringent criterion of MI > 10 was employed. Nested models were compared using the Akaike Information Criteria (AIC); with a lower AIC indicating an optimal model fit. 

#### 2.3.3. Relationship with Health Outcomes

Pearson’s correlation was used to examine the relationship between the scores achieved on the models identified using the EFA and CFA techniques and the scores achieved on the psychological health and connectedness to nature scales. Evidence indicates that exposure to nature and participation in GE can improve psychological health and connectedness to nature [1,3,8]; thus, correlations between the questionnaire scores were expected. Whilst there is relatively less evidence on the role of childhood activities; there is some evidence to suggest that childhood activities influence adult health outcomes [18,20,21]. Scores for the developed scales were calculated by summing scores for items included in the best fitting models. 

## 3. Results

### 3.1. Descriptive Statistics

Descriptive statistics, correlations and residual correlations for all items included in the nature exposure, modified nature exposure and retrospective nature exposure scales are displayed in Table 1 and Table 2. For both scales, the mean score was highest for item one. 

### 3.2. Exploratory Factor Analysis

#### 3.2.1. Nature Exposure Scale.

Inspection of correlation matrices revealed that the average correlation of item one with the remaining items was 0.264, which was below the suggested 0.40 cut-off value; indicating that it was not suitable for EFA (see Table 3). The Cronbach alpha score for the scale was respectable (α = 0.69) but would have been improved by removal of item one. This item was therefore removed from the analysis. 

Following removal, inspection of the correlation matrix revealed that the average correlations of each item with the remaining items were > 0.40 and that the item-total correlations were > 0.30. The Cronbach alpha score for the scale was respectable (α = 0.71). 

The KMO was 0.67 indicating a favourable common variance for factor analysis. Bartlett’s test of sphericity (x^2^ (3) = 222.80, *p* < 0.001) also indicated that the correlation matrix was favourable. The results of analysis revealed that the Eigen value for the single factor was greater than one (1.920) and explained 64.0% of the variance. All three items had excellent loadings on this factor (Table 4). 

#### 3.2.2. Modified Nature Exposure Scale (NES-II)

Inspection of correlation matrices revealed that the average inter-item correlation of item one, was below the suggested 0.40 cut-off value (0.270); indicating that it was not suitable for EFA (Table 5). The Cronbach alpha score for the scale was very good (α = 0.80) but would have been improved by removal of item one. This item was therefore removed from the analysis. 

Following removal, inspection of the correlation matrix revealed that the average correlations of each item with the remaining items were > 0.40 and that the item-total correlations were > 0.30. The Cronbach alpha score for the scale was very good (α = 0.814) and would not have been improved by removal of any of the items. The five remaining items were therefore submitted for EFA. 

The KMO measure of sampling adequacy was 0.74 indicating that the items on the NES-II had adequate common variance for factor analysis. Bartlett’s test of sphericity (x^2^ (10) = 645.109; *p* < 0.001) indicated that the correlation matrix was also favourable. The results of the analysis revealed one factor with an Eigen value greater than one (2.870), which explained 57.4% of the variance. Inspection of the scree plot showed a steep cut-off between the first and second factors. All five items had at excellent loadings on this factor (Table 6). 

#### 3.2.3. Retrospective Nature Exposure Scale (RNES-II)

Inspection of correlation matrices revealed that the average correlations of each item with the remaining items were > 0.40 and that the item-total correlations were > 0.30 (Table 7). The Cronbach alpha score for the scale was very good (α = 0.855) but would have been marginally be improved by the removal of item one (α = 0.857). All items were therefore submitted to EFA.

The KMO measure of sampling adequacy was 0.74 indicating that the items on the RNES-II had adequate common variance for factor analysis. Bartlett’s test of sphericity (x^2^ (10) = 1133.07; *p* < 0.001) indicated that the correlation matrix was also favourable. The results of the analysis revealed one factor with an eigen value of 3.510; explaining 58.5% of the variance. Inspection of the scree plot showed a sharp cut-off between the first and second factors. A one factor model was therefore retained. All items had at least very good loadings on this factor (Table 8). When item one was removed, the results also revealed one factor, with an eigen value of 3.191 (accounting for 63.8% of the variance) and excellent factor loadings (Table 8)

### 3.3. Confirmatory Factor Analysis

#### 3.3.1. Nature Exposure Scale (NES)

Fit indices for the models are displayed in Table 9. Fit indices for the two-factor model of the original NES (model 1) indicated poor model fit. Modification indices were consulted but resulted in an untestable model. Fit indices for the one factor four item model (model 2) were also poor. Modification indices were consulted but made little theoretical sense. Testing of the three-item one factor model resulting from EFA revealed that it had zero degrees of freedom and was therefore untestable.

#### 3.3.2. Modified Nature Exposure Scale (NES-II)

Fit indices for the one factor six item model (model 3) indicated a poor data fit. Modification indices were consulted to free error covariance between items #1 and #2; and #3 and #5 resulting in an improved model fit. However, this model did not meet the criteria on a number of goodness of fit statistics (model 3a) 

**Figure 1:**
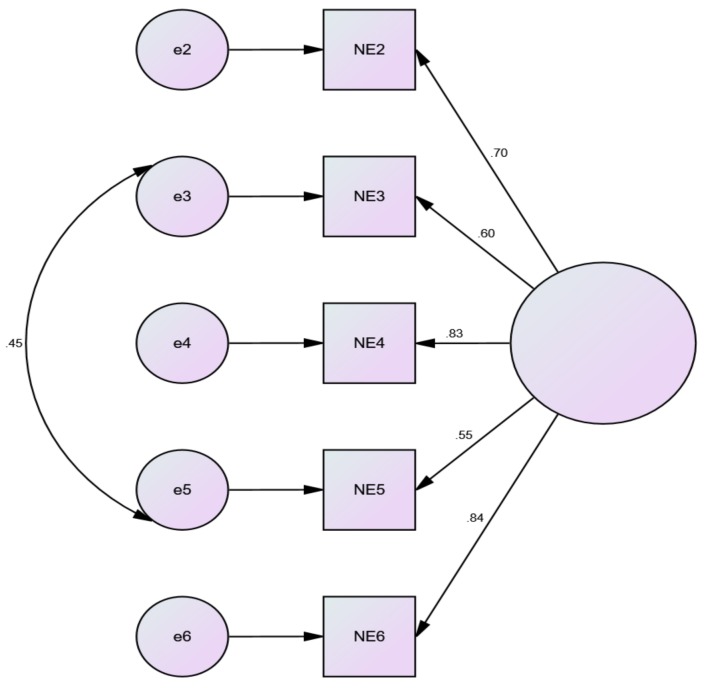
Model fit for the 5-item modified nature exposure scale (NES-II).

Fit indices for the one factor five item model (model 4) supported by the EFA were poor. Modification indices were consulted to free error covariance between items #3 and #5 resulting in a good model fit. The factor loadings and structure of this model (model 4a) can be found in Figure 1. The mean (±SD) score for this final model was 18.43 ± 4.53.

#### 3.3.3. Retrospective Nature Exposure Scale (RNES-II)

**Figure 2:**
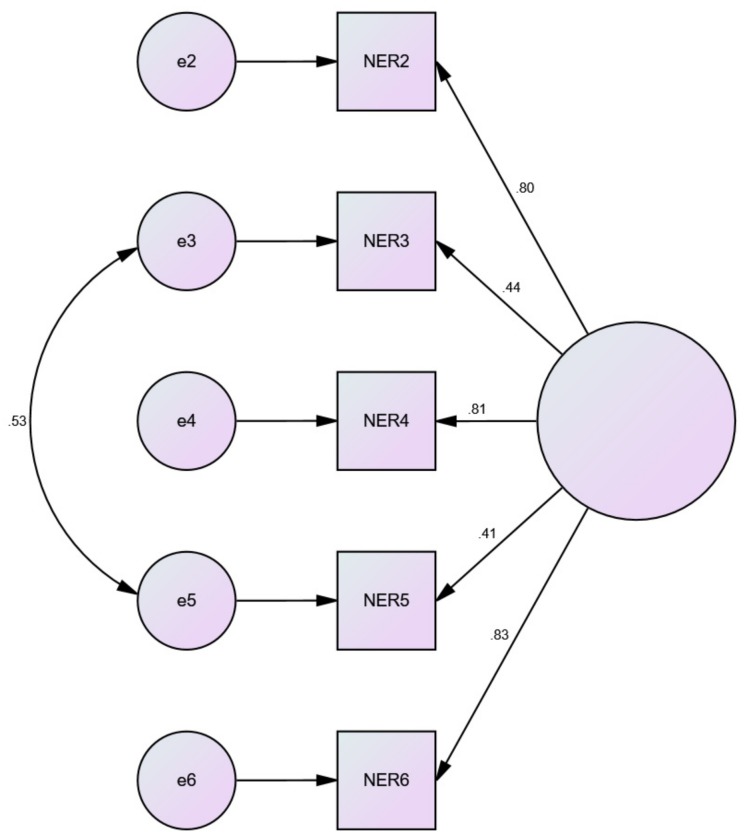
Model fit for the 5-item retrospective nature exposure scale (RNES-II).

Fit indices for the one factor six item model indicated poor data fit (model 5). Modification indices were consulted to free error covariance between items #1 and #3; #1 and #5, and #3 and #5 resulting in an improved model fit (model 5a). However, this model did not meet the criteria for all of the assessed goodness of fit statistics. Fit indices for the one factor five item model (model 6) were also poor. The use of modification indices to free error covariance between items #3 and #5 resulted in an improved model that met the criteria for the majority of goodness of fit statistics (model 6a). This model was also favourable over the 6-item version. The factor loadings and structure of this model can be found in Figure 2. The mean (± SD) score for this final model was 18.67 ± 4.20.

### 3.4. Relationship with Health Outcomes

Pearson’s correlation revealed a significant positive relationship between scores on the 5-item NES-II (model 4) and RNES-II scale (r = 0.533; *p* < 0.001). Pearson’s correlation also revealed significant relationships between the scores on the 5-item NES-II and the psychological health and connectedness to nature measures; in the expected direction (*p* < 0.05) (Table 10). There were also significant relationships between scores on the 5-item RNES-II and the psychological health and connectedness to nature measures (*p* < 0.05).

## 4. Discussion

The aim of this study was to examine the factor structure and reliability of the NES [19], a modified version of the scale to include consideration of nature exposure during PA (NES-II) and a retrospective version of the NES (RNES-II). Preliminary analysis revealed that the item assessing exposure to nature during everyday environments was not suitable for EFA on the NES and NES-II. Whilst it was marginally suitable on the RNES-II; findings were improved following its removal. Exposure to nature during everyday environments might fall outside of an individual’s immediate control. For example, there is evidence to suggest that individuals with a low SES have less nature close to the home [35]. Whilst they may express a desire to have access to these environments, it may not be possible for them to do so. Additionally, whilst an individual might seek a work location in an area surrounded by nature, the need for a regular income would be a key factor in the acceptance of a job. The remaining items on the scales represent a choice to take notice of any surrounding everyday nature and to be exposed and take notice during non-everyday environments. These are actions which individuals are likely to have greater control of and potentially explain why item one does not fit with the other scale items. 

Following removal of item one, EFA demonstrated that the NES and NES-II scales reduced to a single factor representing overall nature exposure. The findings were further confirmed by the CFA; however, the 3-item version of the original NES scale was deemed untestable and the 4-item versions were a poor fit for the data. The purpose of the scale modification was to ensure that assessment of nature exposure includes exposure during PA (GE). The NES-II incorporates this measure; unlike the NES. In addition, the NES-II demonstrated favourable reliability over the NES. The authors therefore recommend that the NES-II is used to assess overall exposure instead of the NES.

For the retrospective scale, EFA revealed a single general factor when both including and excluding item one. However, variables were favourable when item one was excluded. This was also demonstrated by the CFA, where the five-item model provided the best fit for the data. The five item RNES-II should therefore be used to examine retrospective childhood nature exposure. A recent article by the authors examining the health impact of nature exposure across the life course has been published using the NES-II and RNES-II scales [21].

Examination of the relationship between the NES-II and RNES-II scale scores and common health outcomes revealed significant negative correlations with scores achieved for depression, anxiety and stress and positive correlations with scores for connectedness to nature, self-esteem and wellbeing. This supports previous research which has identified improved mental health outcomes in individuals who have greater exposure to nature [1,2,3,7,8,9,11,13,16] and suggest that childhood nature exposure can impact adult health outcomes [18,20,21]. Further exploration of the influence of childhood nature exposure on adult health outcomes and its role in the relationship between current exposure and health is required; as there is a lack of data examining these relationships. 

The main limitation of this study is the use of the online participants, as it may have biased our sample; with only participants who were interested in the topic completing the survey. The use of the prolific tool is likely to have mitigated against the potential problem, as did the use of students from the university who took part for course credits; however, it is still not possible to determine whether the sample is generalizable to the wider population. Further examination of the use of the scales to assess the links between nature exposure and health across the life course are required to further validate their use; as is the test-retest reliability of the scales. 

When considering the current literature on nature exposure; a large body of research is concerned with the importance of nature close to the home [36]. Given the removal of item one from the scales, the item that the captures nature exposure co-incidental to everyday environments; the authors feel that the scales should be renamed to reflect the fact that they capture intentional nature exposure. The authors therefore recommend that these scales be renamed as the ‘intentional nature exposure scale’ and the ‘retrospective intentional nature exposure scale’. The changes have been reflected in the final questionnaire versions displayed in Appendix D and Appendix E. 

## 5. Conclusions

Overall, the findings of the study indicate that the intentional nature exposure scale should be used to assess an individual’s overall level of intentional nature exposure (including GE). In addition, the retrospective intentional nature exposure scale should be used to assess an individual’s exposure during childhood; with future research needing to examine the relative importance of these factors for mental health outcomes.

## Figures and Tables

**Table 1:** Correlation (top right) and residual correlation (bottom left) matrix of the nature exposure scale/modified nature exposure scale with all items, with means and standard deviations.

Item	Item 1	Item 2	Item 3	Item 4	Item 5	Item 6
Item 1	-	0.384	0.278	0.103	0.328	0.190
Item 2	0.036	-	0.416	0.549	0.370	0.538
Item 3	−0.069	−0.159	-	0.477	0.592	0.458
Item 4	−0.245	−0.026	−0.097	-	0.366	0.640
Item 5	−0.011	−0.191	0.032	−0.193	-	0.516
Item 6	−0.179	−0.072	−0.151	0.031	−0.078	-
Mean	2.47	3.52	3.73	4.08	3.44	3.80
SD	1.17	1.13	1.18	1.03	1.28	1.17

Note. All rs significant at 0.01 level. SD = Standard deviation. Residual correlations are derived from a 1-factor exploratory factor analysis (EFA).

**Table 2:** Correlation (top right) and residual correlation (bottom left) matrix of the retrospective nature exposure scale with all items, with means and standard deviations.

Item	Item 1	Item 2	Item 3	Item 4	Item 5	Item 6
Item 1	-	0.506	0.372	0.272	0.455	0.334
Item 2	0.001	-	0.375	0.655	0.356	0.647
Item 3	−0.081	−0.201	-	0.472	0.612	0.426
Item 4	−0.223	0.025	−0.092	-	0.340	0.697
Item 5	0.004	−0.216	−0.099	−0.220	-	0.491
Item 6	−0.183	−0.011	−0.164	0.052	−0.095	-
Mean	3.42	3.47	4.04	3.76	3.99	3.50
*SD*	1.20	1.17	1.07	1.09	1.18	1.19

Note. rs significant at 0.001 level. SD = Standard deviation. Residual correlations are derived from a 1-factor EFA

**Table 3:** Correlation and Cronbach alpha if items deleted for the original nature exposure scale.

Item	Inter-Item Correlation	Item-Total Correlation	α if Item Deleted
Item1	0.264	0.340	0.712
Item 2	0.453	0.625	0.527
Item 3	0.389	0.514	0.599
Item 4	0.342	0.442	0.646

**Table 4:** Factor loadings for 3-item Nature Exposure Scale.

Item	Factor Loading
Item 2	0.804
Item 3	0.772
Item 4	0.823

**Table 5:** Correlation and Cronbach alpha if items deleted for NES-II.

Item	Inter-Item Correlation	Item-Total Correlation	α if Item Deleted
Item1	0.270	0.366	0.811
Item 2	0.449	0.626	0.753
Item 3	0.430	0.604	0.756
Item 4	0.381	0.517	0.778
Item 5	0.427	0.608	0.756
Item 6	0.450	0.629	0.751

**Table 6:** Factor loadings for the 5-item modified Nature Exposure Scale (NES-ii).

Item	Factor Loading
Item 2	0.739
Item 3	0.751
Item 4	0.750
Item 5	0.733
Item 6	0.811

**Table 7:** Correlation and Cronbach alpha if items deleted for the retrospective Nature Exposure Scale (RNES-II).

Item	Inter-Item Correlation	Item-Total Correlation	α if Item Deleted
Item1	0.402	0.506	0.857
Item 2	0.535	0.705	0.819
Item 3	0.489	0.627	0.834
Item 4	0.521	0.679	0.825
Item 5	0.489	0.628	0.834
Item 6	0.550	0.722	0.815

**Table 8:** Factor loadings for 6-item and 5-item the RNES-II.

Item	6-Item	5-Item
Item 1	0.630	
Item 2	0.808	0.787
Item 3	0.749	0.759
Item 4	0.803	0.839
Item 5	0.746	0.735
Item 6	0.835	0.867

Note: Factor loadings taken from pattern matrix output

**Table 9:** Model fit indices.

Model	Description	χ^2^ (df)	χ^2^_Normed_	RMSEA (90%CI)	SRMR	CFI	AIC
1	Original 4 item NES with the hypothesized two factor structure assessing nature exposure in everyday and non-everyday environments.	15.03(1)	15.03	0.191(0.114–0.282)	0.045	0.959	41.03
2	Original 4 item NES with a one factor structure assessing overall nature exposure	32.45(2)	16.223	0.199(0.143–0.262)	0.053	0.911	56.45
3	Modified 6 item NES with a one factor structure assessing overall nature exposure	129.53(9)	14.392	0.187(0.159−0.216)	0.075	0.862	165.53
3a	Modified 6 item NES with a one factor structure assessing overall nature exposure. Modification indices resulted in freeing of error covariance between items #1 and #2 and #3 and #5.	35.82(7)	5.117	0.104(0.072–0.138)	0.051	0.967	75.82
4	Modified 5 item NES with a one factor structure assessing overall nature exposure.	80.82(5)	16.163	0.199(0.162–0.238)	0.067	0.905	110.82
4a	Modified 5 item NES with a one factor structure assessing overall nature exposure. Modification indices resulted in freeing of error covariance between items #3 and #5.	5.34 (4)	1.334	0.030(0.00–0.087)	0.013	0.998	37.34
5	Retrospective 6-item NES with a one factor structure assessing overall nature exposure during childhood	213.93(9)	23.770	0.244(0.216–0.273)	0.109	0.780	249.99
5a	Retrospective 6-item NES with a one factor structure assessing overall nature exposure during childhood. Modification indices resulted in freeing of error covariance between items #1 and #3, #4 and #5, #2 and #3, #2 and #5 and #3 and #5.	58.18(6)	9.697	0.151(0.117–0.187)	0.038	0.944	100.18
6	Retrospective 5-item NES with a one factor structure assessing overall nature exposure during childhood	138.18(5)	27.637	0.264(0.227–0.303)	0.106	0.829	168.18
6a	Retrospective 5-item NES with a one factor structure assessing overall nature exposure during childhood. Modification indices resulted in freeing of error covariance between items #3 and #5.	18.52(4)	4.630	0.097(0.055–0.144)	0.023	0.981	50.520

**Table 10:** Relationship between NES-II and RNES-II scores and psychological health and connectedness to nature variables. DASS: Depression, Anxiety and Stress Scale.

Measure		Mean ± SD	5-Item NES-II		5-Item RNES-II	
			r Value	p Value	r Value	p Value
DASS Scale	Depression	6.84 ± 5.58	−0.322	< 0.001	−0.238	< 0.001
	Anxiety	4.95 ± 4.39	−0.216	< 0.001	−0.170	0.003
	Stress	7.81 ± 4.86	−0.220	< 0.001	−0.143	0.013
Wellbeing		23.67 ± 4.85	0.264	< 0.001	0.201	< 0.001
Self-esteem		27.79 ± 5.76	0.245	< 0.001	0.191	< 0.001
Connectedness to Nature		47.81 ± 8.67	0.491	< 0.001	0.344	< 0.000

## Appendix A: Nature Exposure Scale (Francis, 2011)

We are interested in your exposure to nature, both in your everyday life and activities, and when you take yourself on excursions outside of your everyday environments. We are also interested in your use of natural environments for physical activity. Please complete the following questions to reflect your ***current*** level of exposure to natural environments and participation in physical activity within these environments.

These ‘natural environments’ could be in urban (city parks for example) or rural areas. They could include things such as plants and animals (native or non-native), natural geography (e.g., hills, mountains, deserts, beaches, marshlands), natural water courses and waterscapes (e.g., rivers, streams, lakes, ponds and ocean). Having a view which includes these types of natural environments is also relevant. This is in contrast to the so called ‘built environment’ of houses, buildings, roads and all other such structures created by humans.

### Nature Exposure in Your Everyday Life and Environments

**Item 1:** In your everyday home, travel and work environments and activities, please rate your level of exposure to natural environments (please circle a number)

5	4	3	2	1
High		Medium		Low
Most of my everyday		About half of my everyday		Very little of my everyday
environment is natural		environment is natural		environment is natural

**Item 2:** How much do you notice the natural environments in your everyday life (please circle a number)?

5	4	3	2	1
A great deal		Somewhat		Not much

### Nature Exposure during Excursions OUTSIDE of Everyday Life Environments

These questions relate to your level of exposure to nature when you are outside your everyday environments. This would include trips you make in your leisure time (or occasionally as part of your study, work or social activities) to nature-rich environments in urban, rural or wilderness areas. These might be places that you travel to once a week, or less frequently, either for the express purpose of being in the natural environment or for some other main purpose.

**Item 3:** Please rate the frequency (how often) of exposure to nature-rich environments outside your everyday environment (please circle a number)

5	4	3	2	1
High		Medium		Low
Once a month or less	Once every 6 months	Once a year or less		more often

**Item 4:** How much notice would you take of the nature in these environments (please circle a number)?

5	4	3	2	1
A great deal		Somewhat		Not much

## Appendix B: Nature Exposure Scale II (NES-II)

We are interested in your exposure to nature, both in your everyday life and activities, and when you take yourself on excursions outside of your everyday environments. We are also interested in your use of natural environments for physical activity. Please complete the following questions to reflect your ***current*** level of exposure to natural environments and participation in physical activity within these environments.

These ‘natural environments’ could be in urban (city parks for example) or rural areas. They could include things such as plants and animals (native or non-native), natural geography (e.g., hills, mountains, deserts, beaches, marshlands), natural water courses and waterscapes (e.g., rivers, streams, lakes, ponds and ocean). Having a view which includes these types of natural environments is also relevant. This is in contrast to the so called ‘built environment’ of houses, buildings, roads and all other such structures created by humans.

### Nature Exposure in Your Everyday Life and Environments

**Item 1:** In your everyday home, travel and work environments and activities, please rate your level of exposure to natural environments (please circle a number)

5	4	3	2	1
High		Medium		Low
Most of my everyday		About half of my everyday		Very little of my everyday
environment is natural		environment is natural		environment is natural

**Item 2:** How much do you notice the natural environments in your everyday life (please circle a number)?

5	4	3	2	1
A great deal		Somewhat		Not much

### Nature Exposure during Excursions OUTSIDE of Everyday Life Environments

These questions relate to your level of exposure to nature when you are outside your everyday environments. This would include trips you make in your leisure time (or occasionally as part of your study, work or social activities) to nature-rich environments in urban, rural or wilderness areas. These might be places that you travel to once a week, or less frequently, either for the express purpose of being in the natural environment or for some other main purpose.

**Item 3:** Please rate the frequency (how often) of exposure to nature-rich environments outside your everyday environment (please circle a number)

5	4	3	2	1
High		Medium		Low
Once a month or less	Once every 6 months	Once a year or less		more often

**Item 4:** How much notice would you take of the nature in these environments (please circle a number)?

5	4	3	2	1
A great deal		Somewhat		Not much

### Nature Exposure during Physical Activity

These questions relate to your level of exposure to nature when you are engaging in physical activity. Physical activity in natural environment (called Green Exercise) might include activities such as walking, gardening, fishing, jogging or cycling. These physical activities could be conducted as part of, or coincidental to an everyday activity, or be a planned period of exercise. They might take place in urban, rural or wilderness areas.

**Item 5:** Please rate the frequency (how often) in which you perform physical activity in nature-rich environments (please circle a number)

5	4	3	2	1
High		Medium		Low
Weekly		Once every 6 months		Once a year or less

**Item 6:** How much notice would you take of the nature when you are performing physical activity (please circle a number)?

5	4	3	2	1
A great deal		Somewhat		Not much

## Appendix C: Retrospective Nature Exposure Scale II (RNES-11)

We are interested in your exposure to nature during childhood, both in your everyday life and activities, and when you went on excursions outside of your everyday environments. We are also interested in whether you used natural environments for physical activity. Please complete the following questions to reflect your level of exposure to natural physical environments and participation in physical activity within these environments during childhood. Childhood is considered to take place between the ages of 5-10years.

These ‘natural environments’ could have been in an urban (city parks for example) or rural areas. They could have included things such as plants and animals (native or non-native), natural geography (e.g., hills, mountains, deserts, beaches, marshlands), natural water courses and waterscapes (e.g., rivers, streams, lakes, ponds and ocean). Having had a view which included these types of natural environments is also relevant. This is in contrast to the so called ‘built environment’ of houses, buildings, roads and all other such structures created by humans.

### Nature Exposure in Your Everyday Life and Environments

**Item 1:** In your everyday home, travel and schools’ environments and activities, please rate your level of exposure to natural environments during childhood (please circle a number)

5	4	3	2	1
High		Medium		Low
Most of my everyday		About half of my everyday		Very little of my everyday
everyday environment was natural		environment was natural		environment was natural

**Item 2:** How much did you notice the natural environments in your everyday life (please circle a number)?

5	4	3	2	1
A great deal		Somewhat		Not much

### Nature Exposure during Excursions OUTSIDE of Everyday Life Environments

These questions relate to your level of exposure to nature when you were outside your everyday environments. This would include trips you made in your leisure time (or occasionally as part of your school or family activities) to nature-rich environments in urban, rural or wilderness areas. These might be places that you travelled to once a week, or less frequently, either for the express purpose of being in the natural environment or for some other main purpose.

**Item 3:** Please rate the frequency (how often) of exposure to nature-rich environments outside your everyday environment during childhood (please circle a number)

5	4	3	2	1
High		Medium		Low
Once a month or less	Once every 6 months	Once a year or less		more often

**Item 4:** How much notice did you take of the nature in these environments (please circle a number)?

5	4	3	2	1
A great deal		Somewhat		Not much

### Nature Exposure during Physical Activity

These questions relate to your level of exposure to nature when you engaged in physical activity during your childhood. Physical activity in natural environment (called Green Exercise) might include activities such as walking, gardening, fishing, jogging, cycling or playing. These physical activities could have been conducted as part of, or coincidental to your everyday activities (e.g., school) or have been planned periods of exercise. They might have taken place in urban, rural or wilderness areas.

**Item 5**: Please rate the frequency (how often) in which you performed physical activity in nature-rich environments during childhood (please circle a number)

5	4	3	2	1
High		Medium		Low
Weekly		Once every 6 months		Once a year or less

**Item 6:** How much notice did you take of the nature when you were performing physical activity (please circle a number)?

5	4	3	2	1
A great deal		Somewhat		Not much

## Appendix D: Intentional Nature Exposure Scale (INES)

We are interested in your exposure to nature, both in your everyday life and activities, and when you take yourself on excursions outside of your everyday environments. We are also interested in your use of natural environments for physical activity. Please complete the following questions to reflect your current level of exposure to natural environments and participation in physical activity within these environments.

These ‘natural environments’ could be in urban (city parks for example) or rural areas. They could include things such as plants and animals (native or non-native), natural geography (e.g., hills, mountains, deserts, beaches, marshlands), natural water courses and waterscapes (e.g., rivers, streams, lakes, ponds and ocean). Having a view which includes these types of natural environments is also relevant. This is in contrast to the so called ‘built environment’ of houses, buildings, roads and all other such structures created by humans.

### Nature Exposure in Your Everyday Life and Environments

How much do you notice the natural environments in your everyday life (please circle a number)?

5	4	3	2	1
A great deal		Somewhat		Not much

### Nature Exposure during Excursions OUTSIDE of Everyday Life Environments

These questions relate to your level of exposure to nature when you are outside your everyday environments. This would include trips you make in your leisure time (or occasionally as part of your study, work or social activities) to nature-rich environments in urban, rural or wilderness areas. These might be places that you travel to once a week, or less frequently, either for the express purpose of being in the natural environment or for some other main purpose.

Please rate the frequency (how often) of exposure to nature-rich environments outside your everyday environment (please circle a number)

5	4	3	2	1
High		Medium		Low
Once a month or less	Once every 6 months	Once a year or less		more often

How much notice would you take of the nature in these environments (please circle a number)?

5	4	3	2	1
A great deal		Somewhat		Not much

### Nature Exposure during Physical Activity

These questions relate to your level of exposure to nature when you are engaging in physical activity. Physical activity in natural environment (called Green Exercise) might include activities such as walking, gardening, fishing, jogging or cycling. These physical activities could be conducted as part of, or coincidental to an everyday activity, or be a planned period of exercise. They might take place in urban, rural or wilderness areas.

Please rate the frequency (how often) in which you perform physical activity in nature-rich environments (please circle a number)

5	4	3	2	1
High		Medium		Low
Weekly		Once every 6 months		Once a year or less

How much notice would you take of the nature when you are performing physical activity (please circle a number)?

5	4	3	2	1
A great deal		Somewhat		Not much

## Appendix E: Retrospective Intentional Nature Exposure Scale (R-INES)

We are interested in your exposure to nature during childhood, both in your everyday life and activities, and when you went on excursions outside of your everyday environments. We are also interested in whether you used natural environments for physical activity. Please complete the following questions to reflect your level of exposure to natural physical environments and participation in physical activity within these environments during childhood. Childhood is considered to take place between the ages of 5-10years.

These ‘natural environments’ could have been in an urban (city parks for example) or rural areas. They could have included things such as plants and animals (native or non-native), natural geography (e.g., hills, mountains, deserts, beaches, marshlands), natural water courses and waterscapes (e.g., rivers, streams, lakes, ponds and ocean). Having had a view which included these types of natural environments is also relevant. This is in contrast to the so called ‘built environment’ of houses, buildings, roads and all other such structures created by humans.

### Nature Exposure in Your Everyday Life and Environments

How much did you notice the natural environments in your everyday life (please circle a number)?

5	4	3	2	1
A great deal		Somewhat		Not much

### Nature Exposure during Excursions OUTSIDE of Everyday Life Environments

These questions relate to your level of exposure to nature when you were outside your everyday environments. This would include trips you made in your leisure time (or occasionally as part of your school or family activities) to nature-rich environments in urban, rural or wilderness areas. These might be places that you travelled to once a week, or less frequently, either for the express purpose of being in the natural environment or for some other main purpose.

Please rate the frequency (how often) of exposure to nature-rich environments outside your everyday environment during childhood (please circle a number)

5	4	3	2	1
High		Medium		Low
Once a month or less	Once every 6 months	Once a year or less		more often

How much notice did you take of the nature in these environments (please circle a number)?

5	4	3	2	1
A great deal		Somewhat		Not much

### Nature Exposure during Physical Activity

These questions relate to your level of exposure to nature when you engaged in physical activity during your childhood. Physical activity in natural environment (called Green Exercise) might include activities such as walking, gardening, fishing, jogging, cycling or playing. These physical activities could have been conducted as part of, or coincidental to your everyday activities (e.g., school) or have been planned periods of exercise. They might have taken place in urban, rural or wilderness areas.

Please rate the frequency (how often) in which you performed physical activity in nature-rich environments during childhood (please circle a number)

5	4	3	2	1
High		Medium		Low
Weekly		Once every 6 months		Once a year or less

How much notice did you take of the nature when you were performing physical activity (please circle a number)?

5	4	3	2	1
A great deal		Somewhat		Not much
